# The Role of Sports Clothing in Thermoregulation, Comfort, and Performance During Exercise in the Heat: A Narrative Review

**DOI:** 10.1186/s40798-022-00449-4

**Published:** 2022-04-28

**Authors:** Isaiah Di Domenico, Samantha M. Hoffmann, Paul K. Collins

**Affiliations:** 1 Centre for Sport Research (CSR), School of Exercise and Nutrition Sciences, Deakin University, Geelong, VIC Australia; 2 School of Engineering, Deakin University, Geelong, VIC Australia

**Keywords:** Thermoregulation, Clothing comfort, Aerobic exercise, Body cooling, Body temperature, Sportswear, Heat Stress, Evaporation

## Abstract

The aims of this review are to (1) summarise the current research of sports clothing as it relates to thermoregulation, comfort, and performance during exercise in the heat, (2) identify methodological limitations and gaps in the knowledge base of sports clothing, and (3) provide recommendations for exercise testing protocols to accurately assess the impact of sports clothing in athletic populations during exercise in the heat. Sports clothing consists of lightweight and breathable fabrics, surface treatments, and various designs which aim to enhance sweat evaporation and comfort during exercise in the heat. Sports clothing comprised of natural, synthetic, and chemically treated fabrics has been investigated during exercise of varying durations (15–120 min), intensities (20–70% VO_2_ max) and types (fixed intensity, incremental, self-paced), and in an array of climatic conditions (18–40 °C, 20–60% relative humidity). To date, few studies have identified significant differences in thermo-physiological, perceptual, and performance measures between natural and synthetic fabrics or compared the effect of chemical treatments to their non-treated equivalent on such measures during exercise. Collectively, previous wearer trials have failed to replicate the upper limit of training and competition demands when assessing sports clothing in endurance-trained individuals who regularly train and compete in hot and humid climates. Clothing comfort has also been evaluated using simple scales which fail to capture intricate detail pertaining to psychological and sensorial parameters. The incorporation of protocols using hot and humid climates (≥ 30 °C, ≥ 70% relative humidity) and longer exercise durations (> 45 min) is warranted. Future research should also consider exploring the effect of sports clothing on thermal, physiological, perceptual, and performance measures between males and females, and assessing clothing comfort using a multi-dimensional approach.

## Key Points

- Research assessing the effect of sports clothing on thermoregulation, comfort, and performance during exercise in the heat has used protocols characterised by light-to-moderate intensities, short-to-moderate durations, and mild environmental conditions.
- Disparities across methodologies, and insufficient applications of thermal–physiological and perceptual strain, have led to mixed findings concerning the effect of sports clothing to enhance thermoregulation, comfort, and performance in both recreationally active and elite level athletes.
- Future research should continue to focus on developing and assessing new sports clothing designs using protocols which reflect actual training and competition demands to understand which fabric compositions and characteristics may be optimal to maximise thermoregulation, comfort, and performance during exercise in the heat.

## Background

Thermoregulation is the process of regulating the body’s core temperature. During exercise in warm environments, thermoregulation is primarily achieved via sweat evaporation as the body strives to achieve a thermal steady state by balancing metabolic heat production and heat loss [1]. Thermoregulation is particularly important for endurance athletes who often compete in hot and humid environments where the body’s ability to thermoregulate is compromised, increasing their risk of heat illness and having a detrimental impact on performance [2, 3]. Methods to enhance body cooling have received considerable attention in the scientific literature. Focus in this space is warranted as events like the 2021 Summer Olympics in Tokyo were one of the hottest and most humid to date (averages of 32.2 °C and 70% relative humidity [RH]), whilst the 2022 FIFA World Cup in Qatar will likely require athletes to compete in temperatures exceeding 40 °C [2]. There are many cooling options available to athletes such as cooling/ice vests, neck coolers, cold drinks, and ice slurries [2]. However, these options may be difficult to administer during exercise due to practical limitations such as excess weight, skin irritation/discomfort [4], and ingestion issues [5]. Additionally, some of these cooling options are unfeasible to use in events like marathons, long-distance cycling and triathlons due to rules and regulations [6, 7]. Clothing selection presents a unique opportunity to aid thermoregulation during exercise in the heat without the practical limitations of the aforementioned cooling strategies and plays an integral role in comfort; a complex sensation comprised of various sensory inputs including psychological, sensorial, and body-movement factors [8]. By nature, clothing increases insulation which provides a barrier to evaporative heat loss in hot environments [8–10]. However, sports clothing aims to mitigate this insulative effect via breathable and lightweight designs made from synthetic materials that aim to improve moisture transport (i.e. wicking of sweat) to promote evaporative heat loss, maximise wearer comfort, and in turn, enhance performance [11, 12].

Sports Medicine Australia (SMA) acknowledges clothing selection as an important consideration for mitigating heat stress risk during exercise in the heat [13]. SMA’s guidelines indicate that removing unnecessary clothing layers, minimising skin coverage, and selecting lightweight and breathable clothing will improve sweat evaporation and therefore heat dissipation [13]. However, these guidelines lack specificity pertaining to important characteristics of clothing including fabric composition, knit structure, and fit. This lack of specificity may originate from the variation in assessment parameters and methodologies used across many studies in this space which has contributed to a lack of conclusive evidence. Studies assessing the effectiveness of sports clothing to maximise thermoregulation, comfort, and performance have examined a variety of fabrics and textiles including natural fibres [14–16], synthetic fibres [17–19], natural and synthetic fibre blends [16, 20, 21], and chemically treated fibres [22, 23]. The clothing examined is characterised by several material properties that encompass varying levels of insulation, mass, and air permeability, as well as different types of knit structures and fits (i.e. compression vs skin coverage). Additionally, these studies have also used an array of environmental conditions (18–40 °C, 20–60% RH) whilst assessing sports clothing during exercise of varying intensities (20–70% maximal oxygen consumption [VO_2_ max / peak]), durations (15–120 min), and trial types (fixed intensity, incremental, self-paced) in recreationally active and athletic populations. The variation and lack of comparative studies across the current body of the literature has led to much conjecture surrounding the effectiveness and applicability of clothing to keep wearers cool and comfortable, enhance performance, and reduce the risk of heat-related health problems [7]. Indeed, very few studies have measured or linked the ability for clothing to improve exercise performance via related improvements in thermoregulation and comfort. The variation and lack of direct comparisons reported in the literature has likely created difficulties for athletes and coaches in determining ‘best practice’ regarding clothing selection for training and competition.

The purpose of this review is to collate, summarise, and discuss the current body of the literature and its methodological limitations in the assessment of sports clothing to promote thermoregulation, comfort, and performance during exercise in the heat. A subsequent aim is to provide recommendations for exercise testing protocols to accurately assess the impact of sports clothing in athletic populations during exercise in the heat. This review first presents an overview of the fabric and material properties of sports clothing, and a discussion on comfort assessment during exercise. Thereafter, exercise protocols and laboratory conditions administered in the assessment of sports clothing are critically appraised. Finally, implications for future research evaluating the impact of sports clothing during exercise in the heat are presented. To be considered for this review, studies needed to measure thermoregulatory parameters (i.e. core temperature and/or skin temperature) and comfort metrics within the same investigation. A search on PubMed and SPORTDiscus databases was conducted in February 2021 using the terms: ‘clothing’, ‘apparel’, ‘garment’, ‘sportswear’, ‘cooling’, ‘thermoregulation’, ‘comfort’, ‘performance’, ‘wicking’, and ‘evaporation’. Some studies were also sourced from the reference lists of relevant review articles including those by Kicklighter et al. [12] and Davis and Bishop [24]. A total of 51 studies were originally sourced following the literature search, of which 19 were excluded for not assessing thermoregulatory and comfort parameters within the same investigation. Thus, this review has captured 32 individual studies between 1995 and 2020.

## Fibres, Fabric Properties and Material Testing

There are many fabrics used in sports clothing including natural fibres synthesised from plants and animals (e.g. cotton, wool) and synthetic fibres produced through chemical synthesis (e.g. polyester, nylon). Each fabric has its own strengths and limitations pertaining to thermoregulation and comfort which can be affected by its material properties. The following section summarises the most frequently assessed fabric compositions in the current body of the literature, describes their strengths and limitations pertaining to thermoregulation and comfort during exercise, and discusses the role of several material properties in heat dissipation and comfort.

### Natural and Synthetic Fibres

Polyester is the most commonly used synthetic material in sports apparel due to its dimensional stability, smooth feel, and low cost [25]. However, its low moisture absorption may be a limitation during situations of immense sweating as the increased moisture on the skin’s surface may lead to sensations of skin wettedness and discomfort [21]. De Sousa et al. [14] and Roberts et al. [26] compared polyester and cotton sports clothing during exercise. De Sousa et al. [14] noted significant reductions in core temperature, whilst Roberts et al. [26] observed significant improvements in thermal sensation and comfort with polyester. However, the testing conditions used by Roberts et al. [26] included mild and dry climates (20.6 °C, 47.5% RH) and low-to-moderate intensity efforts separated by 70 s rest which may not have induced immense sweating in participants. Other studies have observed no comfort or thermoregulatory benefit from wearing polyester during exercise when compared to cotton [20, 27–29] or wool [16, 30]. These discrepancies may be explained by methodological differences between the studies as environmental conditions (20.6–33 °C, 20–60% RH) and exercise durations varied (30–60 min). Nylon is another synthetic material commonly used in sports clothing which has higher moisture absorption and better wicking capabilities than polyester [25]. However, nylon has a slower drying rate [25] which can induce an undesirable chilling effect post exercise [31]. Clothing wettedness was significantly reduced during exercise when wearing an upper body compression garment comprised of nylon compared to cotton [32]. In contrast, Leoz-Abaurrea et al. [33] and Leoz-Abaurrea and Aguado-Jimenez [34] identified no significant reductions in core temperature, skin temperature, sweat rate, sweat loss or skin/clothing wettedness when comparing nylon and cotton sports clothing despite using similar testing protocols [33] and identical fabric compositions [34]. The studies reporting no significant changes included both male and female subjects [33, 34], compared to the others who included only males [32], suggesting sex as a potential confounder. Cotton is a natural fibre with better moisture absorption capabilities than most synthetic fibres [10], but it can cause skin irritation and undesired stickiness sensations during exercise [25, 35]. Despite this, thermal sensation was significantly improved when wearing a cotton t-shirt during exercise in hot and dry conditions compared to an upper body compression garment composed of nylon [34]. Indeed, some studies have reported significant reductions in core temperature [34, 36], skin temperature [30], sweat loss [30], and heart rate [30, 36] when wearing cotton clothing during exercise compared to synthetic fabrics. Wool is another natural fibre with better wicking capabilities than cotton [25] and is shown to significantly reduce core temperature when compared to polyester during moderate intensity exercise in hot and dry conditions [16]. However, wool is slow-to-dry and can be heavier than synthetic materials [25]. Collectively, these results may suggest that the optimal sports clothing composition for heat dissipation and comfort may vary depending on the environmental conditions, activity duration and intensity, and individual preference.

### Chemically Treated Fabrics

The chemical treatment of fabrics aims to elevate the heat dissipation capacity of sports clothing via enhanced heat energy release and increased wicking to improve comfort and thermoregulation [9]. Phase change material (PCM) is one form of treatment where microcapsules are applied to textile fibres to allow for heat energy absorption and heat energy release. The microcapsules change from liquid to solid states when a specific temperature is reached [37]. Other chemical treatments such as silicone emulsion can enhance the wicking properties of sports clothing [38], whilst titanium dioxide is proposed to enhance thermal conductance and improve thermal comfort [23]. To date, chemically treated fabrics and their thermoregulatory capacities have been primarily assessed in laboratory settings without human participants [39–41]. However, one study by McFarlin et al. [22] compared the effect of a PCM-treated polyester t-shirt to an untreated t-shirt on thermoregulation, comfort, and performance during exercise in hot and humid conditions. Endurance-trained males and females experienced significant reductions in skin temperature and rating of perceived exertion (RPE), and significant increases in comfort and exercise capacity (8%) when wearing the polyester-PCM t-shirt [22]. But given that the assessment of chemically treated fabrics on thermoregulation, comfort, and performance during exercise is limited, future research is needed to confirm these findings and expand current knowledge on the role of chemically treated fabrics in sports clothing.

### Material Properties

The material properties of clothing also impact thermoregulation and comfort including fit, knit structure, air permeability, wicking ability, and insulation. These properties, with the exception of wicking ability, are commonly reported across the current body of the literature but vary due to differences in the clothing designs used (see Table 1). For example, some studies have included full-body clothing designs that cover varying levels of the skin [17, 19], whilst others have used upper and lower body compression clothing [33, 42] as well as short and full-length shirts and pants [36, 43]. Clothing fit is integral to the development of the skin-to-material interface known as the microclimate [7]. Maintaining a stable microclimate temperature and RH is critical to maximising comfort and the rate of evaporative heat loss from the body [11, 44] as microclimate affects thermal comfort, skin temperature, and, in turn, the thermoregulatory response during exercise [10]. Only one study has reported clothing fit and microclimate humidity within the same investigation [29]. Microclimate humidity was significantly reduced during vigorous intensity exercise in hot and dry conditions when a tight-fitting synthetic t-shirt was worn compared to a tight-fitting t-shirt comprised of natural materials [29]. However, a comparison was not made to loose fitting clothing which is important as tight-fitting clothes provide less insulation as the volume of trapped air between the skin and clothing is minimised [8]. Tight-fitting clothes may also impede convective heat loss during exercise via reduced air permeability [9]. Loose-fitted clothing may improve the efficiency of heat dissipation during exercise [7] and improve thermal comfort in moderate heat by increasing air permeability and air flow across the skin’s surface [45]. To date, very few studies have compared the impact of tight-fitted and loose-fitted clothing on thermoregulation, comfort, and performance during exercise in the heat. However, Barwood et al. [42] and MacRae et al. [46] have analysed the effects of oversized and correctly fitted compression garments on thermoregulation and comfort [42, 46]. Whilst fitted and oversized compression garments induced significant increases in skin temperature [42, 46], and negative effects on thermal comfort and thermal sensation [46] compared to exercise shorts, no significant differences were observed between oversized and fitted garments. The negative effects of the compression garments during exercise could be the result of greater body coverage [46] as more of the skin's surface is covered by material which can have a negative impact on heat transfer from the body to the environment [47, 48]. In contrast, the lack of significant differences between the oversized and correctly fitted garments may be explained by similar amounts of pressure exerted by the compression garments [42, 46] given they were both considered to be tight-fitting. Clearly, future research is needed to compare the effect of tight-fitted and loose-fitted clothing on microclimate, thermoregulation, and overall comfort. Such work could generate clothing recommendations pertaining to fit and skin coverage for athletic and recreationally active populations who exercise in specific environmental conditions.

**Table 1:** Summary of material properties from sports apparel designed to enhance thermoregulation, performance, and comfort

Study	Apparel	Fabric Composition	Structure	Fit	Insulation (clo)	Mass (g^.^m^−2^)	Air permeability (mm/s)	Water vapour permeability (g^.^m^−2^)
Abdallah et al. [17]	Long sleeve shirt and pants	CTR (80% nylon, 20% spandex) EXP (78% nylon, 22% spandex)	CTR: warp EXP: warp	Form fitting	–	CTR: 195 EXP: 160	–	–
Barwood et al. [42	Lower-body compression garment	–	–	Form fitting	–	–	–	–
Brazaitis et al. [27]	Long sleeve t-shirt	CTR (94% cotton, 6% elastane) EXP (93% polyester, 7% elastane)	–	–	–	CTR: 186 EXP: 183	CTR: 327.1 EXP: 528.1	CTR: 6134.2 EXP: 5793.8
Collins et al. [55]	Short sleeve and sleeveless t-shirts	CTR (85–100% polyester and 90–100% cotton) EXP (100% polyester)	–	–	–	–	–	–
Corbett et al. [19]	Combination of shorts, short sleeve jerseys, long sleeve jerseys, cycling jacket and skull cap	CTR (shorts, 74% nylon, 26% elastane) EXP1 (shorts, 78% nylon, 22% elastane; short sleeve jersey, 100% polyester) EXP2 (shorts, 75% polyamide, 22% elastane, 3% carbon fibre; short sleeve jersey, 54% polyamide, 36% polyester, 7% elastane, 3% lyocell) EXP3 (long sleeve base layer, 84% polyester, 16% elastane; full length pants, 100% nylon; cycling jacket, 100% nylon; skull cap (100% polyester)	–	–	–	–	–	–
Davis et al. [20]	Short sleeve t-shirt	CTR (100% cotton) EXP1 (100% polyester) EXP2 (50% soy-bean, 50% cotton)	CTR: knit EXP1: knit EXP2: knit	-	-	CTR: 143 EXP1: 130 EXP2: 215	-	-
De Sousa et al. [14]	Short sleeve t-shirt	CTR (100% cotton) EXP (81% polyester, 19% elastane)	–	Form fitting	–	–	–	–
Filingeri et al. [60]	Short sleeve t-shirt	CTR (dry) EXP (wetted)	–	Form fitting	CTR: 0.20 EXP: 0.26	CTR: 101 EXP: 154	–	–
Gavin et al. [28]	Short sleeve t-shirt and shorts	CTR (cotton) EXP1 (synthetic material) EXP2 (lycra)	–	Form fitting	CTR: 0.28 EXP1: 0.27 EXP2: 0.09	–	–	–
Gonzales et al. [49]	Short sleeve jersey	(100% polyester) CTR (2 mm knit size) EXP1 (3 mm knit size) EXP2 (3.5 mm knit size)	CTR: knit EXP1: knit EXP2: knit	–	–	CTR: 171 EXP1: 160 EXP2: 120	–	–
Ha et al. [43]	Long sleeve shirt and full-length pants	CTR (100% polyester, low absorption, low air permeability) EXP1 (100% polyester, low absorption, high air permeability) EXP2 (100% cotton, high absorption, high air permeability)	–	–	CTR: 0.65 EXP1: 0.73 EXP2: 0.73	CTR: 174.6 EXP1: 169.1 EXP2: 189.4	CTR: 40.1 EXP1: 1200 EXP2: 1080	–
Ha et al. [36]	Short sleeve t-shirt, long-sleeved working dress and full-length pants	CTR (100% cotton) EXP (100% polyester)	–	–	CTR: 1.24 EXP: 1.25	CTR: 275.3 EXP: 290.8	–	–
Herten et al. [51]	Shirt	CTR (100% polyester) EXP (67% polyester, 33% lyocell)	–	Form fitting	–	CTR: 153.2 EXP: 160.8	CTR: 925.2 EXP: 1212.6	–
Kaplan et al. [21]	Long sleeve t-shirt	CTR (100% cotton) EXP1 (100% polyester) EXP2 (100% polyester) EXP3 (70% cotton, 30% polyester) EXP4 (95% modified polyester, 5% elastane)	CTR: woven EXP1: interlock EXP2: interlock EXP3: interlock EXP4: woven	–	–	CTR: 166.5 EXP1: 189.3 EXP2: 183.6 EXP3: 459.9 EXP4: 242.9	CTR: 468 EXP1: 1854 EXP2: 1317 EXP3: 399 EXP4: 210	–
Kwon et al. [30]	Long sleeve t-shirt and full-length pants	CTR (100% cotton) EXP1 (50% cotton, 50% wool) EXP2 (100% polyester)	–	–	–	CTR: 125.2 EXP1: 120.8 EXP2: 127.2	–	–
Laing et al. [16]	Long sleeve upper body garment	CTR (100% polyester) EXP1 (52% wool, 48% polyester) EXP2 (100% wool)	CTR: interlock EXP1: plated EXP2: woven	–	–	CTR: 229.7 EXP1: 206.6 EXP2: 197.2	–	–
Leoz-Abaurrea et al. [33]	Short sleeve compression garment and t-shirt	CTR (100% cotton) EXP (94% nylon, 4% elastane, 2% polypropylene)	–	Form fitting	–	–	–	–
Leoz-Abaurrea et al. [32]	Short sleeve compression garment and t-shirt	CTR (100% cotton) EXP (94% nylon, 4% elastane, 2% polypropylene)	–	Form fitting	–	–	–	–
Leoz-Abaurrea et al. [34]	Short sleeve compression garment and t-shirt	CTR (100% cotton) EXP (94% nylon, 4% elastane, 2% polypropylene)	–	Form fitting	–	–	–	–
Lin et al. [63]	Sports bra	(35% polyester, 8% lycra, 50% cotton) CTR (single layer) EXP (dynamic water pumping fabrics)	CTR: woven EXP: woven tuck stitch	–	CTR: 0.18 EXP: 0.18	CTR: 287 EXP: 323	–	CTR: 972.8 EXP: 1051.8
MacRae et al. [46]	Full body compression garment and shorts	(76% nylon, 24% elastane) CTR (shorts) EXP1 (correct size) EXP2 (oversized)	EXP1: knit EXP2: knit	Form fitting	–	EXP1: 195 EXP2: 195	EXP1: 810 EXP2: 810	–
McFarlin et al. [22]	T-shirt	CTR (polyester blend) EXP (polyester + 30% PCM)	–	–	–	–	–	–
Raccuglia et al. [56]	Short sleeve t-shirt	(100% polyester) CTR (92.7% sample contact) EXP1 (87.5% sample contact) EXP2 (66.3% sample contact)	–	Loose fitting	–	CTR: 85 EXP1: 85 EXP2: 80	–	–
Roberts et al. [26]	T-shirt	CTR (bare chested) EXP1 (85% polyester, 15% elastane) EXP2 (65% nylon, 21% polyester, 14% lycra) EXP3 (100% cotton)	CTR: mesh EXP1: mesh EXP2: mesh	–	–	–	–	–
Scholler et al. [18]	6 synthetic full body racing suits	EXP1 (59% polyester, 25% polyamide, 16% elastane) EXP2 (82% polyester, 18% elastane) EXP3 (59% polyamide, 26% polyester, 15% elastane) EXP4 (58% polyamide, 26% polyester, 16% elastane) EXP5 (81% polyester, 19% elastane) EXP6 (80% polyester, 20% elastane)	EXP1: knit EXP2: woven EXP3: knit EXP4: knit EXP5: woven EXP6: woven	–	–	EXP1: 88 EXP2:112 EXP3:136 EXP4:132 EXP5:136 EXP6:140	–	–
Sperlich et al. [29]	t-shirt	CTR (100% cotton) EXP1 (91% polyester, 9% spandex, 4 channel fibres) EXP2 (100% polyester, 6 channel fibres) EXP3 (90% polyester, 10% spandex, 8 channel fibres)	–	Form fitting	–	CTR: 185 EXP1: 130 EXP2: 125 EXP3: 148	–	–
Tsuji et al. [61]	Long sleeve t-shirt and shorts	CTR (shorts, 100% polyester) EXP (long sleeve shirt, 55% polyester, 45% cotton; shorts, 100% polyester)	–	–	–	–	–	–
Ueda et al. [15]	Long sleeve t-shirts	(100% cotton)	CTR: knit EXP1: stockinet EXP2: knit EXP3: jacquard EXP4: mesh	–	–	CTR: 233 EXP1: 133 EXP2: 182 EXP3: 131 EXP4: 171	–	–
Varadaraju et al. [50]	Short sleeve t-shirt	CTR (polyester) EXP1 (polyester, mesh sides) EXP2 (polyester, mesh sides, and chest) EXP3 (polyester, mesh sides, chest, and back)	CTR: tricot warp knit EXP1: open warp knit EXP2: open warp knit EXP3: open warp knit	–	–	CTR: 125 EXP1: 120 EXP2: 110 EXP3: 115	CTR: 1630 EXP1: 4000 EXP2: 4000 EXP3: 4000	CTR: 1140 EXP1: 1481 EXP2: 1481 EXP3: 1481
Wang et al. [77]	Upper body garment	CTR (cotton) EXP1 (body mapping sportswear) EXP2 (body mapping sportswear with face and back mesh designs)	–	Form fitting	CTR: 0.19 EXP1: 0.18 EXP2: 0.16	–	CTR: 387.7 EXP1: 723.5 EXP2: –	–
Wingo et al. [81]	Long sleeve t-shirt	CTR (bare chested) EXP1 (synthetic) EXP2 (cotton)	–	–	–	–	–	–
Zhang et al. [45]	Short sleeve t-shirts	(100% polyester) Nine shirts differing in neck and hem opening size	Interlock	–	–	152	–	–

*CTR* control, *EXP* experimental

The effect of other material properties such as knit structure, air permeability, and insulation on thermoregulation and comfort during exercise is well understood. Knit structure influences heat transfer and provides an opportunity to enhance clothing comfort depending on the patterns used [10, 11]. Stockinet and mesh knit structures improved comfort during exercise when compared to plain knit and jacquard knit structures in cotton t-shirts [15]. Large knit sizes and open warp knit structures significantly reduced skin temperature [49, 50] and microclimate humidity [50] as well as improved thermal sensation [49] and comfort [50] in polyester clothing. Additionally, sweat absorption and thermal sensation were significantly improved in weaved cycling clothes compared to knitted cycling clothes during exercise, suggesting fabric construction plays an important role in managing thermal sensation and the transfer of heat from the skin to the environment [18]. Air permeability is dependent on fabric structure [10] as improved air permeability enhances heat exchange between the body and the environment via convection and evaporation [35]. Brazaitis et al. [27] determined that synthetic t-shirts with superior air permeability significantly increased sweat evaporation during exercise when compared to t-shirts made of natural fabrics harnessing lower air permeability. Kaplan and Okur [21] assessed the effect of five different t-shirts on comfort during exercise, with each shirt having varying levels of air permeability (see Table 1). Thermal comfort ratings were poorer (p < 0.05) when the t-shirt with the lowest air permeability was used compared to all other t-shirts [21]. A similar interaction was observed by Herten et al. [51] with results that approached statistical significance (p > 0.06). Herten et al. [51] noted lower microclimate humidity (10%) following 15 min of walking during a 60-min trial when a shirt with higher air permeability was worn compared to a shirt with lower air permeability. Taken together, these results suggest that greater air permeability may decrease the insulative effect of clothing, thereby reducing the barrier to evaporative heat loss in hot environments.

### Material Testing and Evaporative Heat Loss Capacity

Assessing the ecological validity of sports clothing requires human trials that mimic actual conditions of the clothing’s intended use [9, 10]. However, material testing using specialised equipment such as sweating guarded hotplates and thermal manikins can inform the plausibility and rationale for wearer trials. The material properties previously discussed have the potential to influence thermoregulation and comfort across environmental conditions. However, the evaporative heat loss capacity and wicking ability of fabrics used in the current body of the literature are not reported which is critical given that sweat wicking and evaporation are the primary mechanisms of heat loss during exercise in hot conditions [1, 8]. Moreover, these parameters can be easily determined using appropriate tests such as sweating guarded-hotplate tests and vertical wicking tests [52, 53]. Sweat evaporation is proportional to the water vapour permeability of clothing; another material property reported to play a critical role in overall wearer comfort [11]. Measuring these parameters allows for a clearer comparison and validation of results between material testing and human trials, yet only one study to date by Jiao et al. [54] has reported these material properties when testing sports clothing during exercise. Jiao et al. [54] observed the effect of three different marathon clothing designs on thermoregulation and performance in elite athletes during prolonged fixed-intensity exercise (45 min) in hot and humid laboratory conditions (30 °C, 50% RH). Whilst no significant performance differences were observed, the clothing design with the highest water vapour permeability and heat dissipation capacity induced significant reductions in core temperature and skin temperature, and significant improvements in sweat evaporation efficiency [54]. Clearly, these results may have been expected given the clothing’s material properties. However, the reinforcement given by the wearer trials provides the ultimate validation and confirms the potential shown by the clothing during material testing. This level of confirmation between material testing and wearer trials is limited in the current body of the literature. Approximately 40% of the studies in this review reported very few or typical material properties (i.e. mass, insulation, fit) and generated results from wearer trials that do not reinforce the positive effects proposed by the material properties [15, 17, 20, 28, 29, 32–34, 42, 51, 55, 56].

## The Assessment of Clothing Comfort

According to Ozdil and Anand [25], clothing comfort comprises four main considerations:

1. psychological; concerning the design features of apparel,
2. sensorial; concerning the mechanical sensations caused by the fabric including softness and clinginess,
3. body-movement; concerning the fit of the clothing and the freedom of movement it allows, and
4. thermo-physiological; concerning moisture and air permeability and any aspect that directly influences the wearer’s thermoregulation such as wicking.

**Fig. 1:**
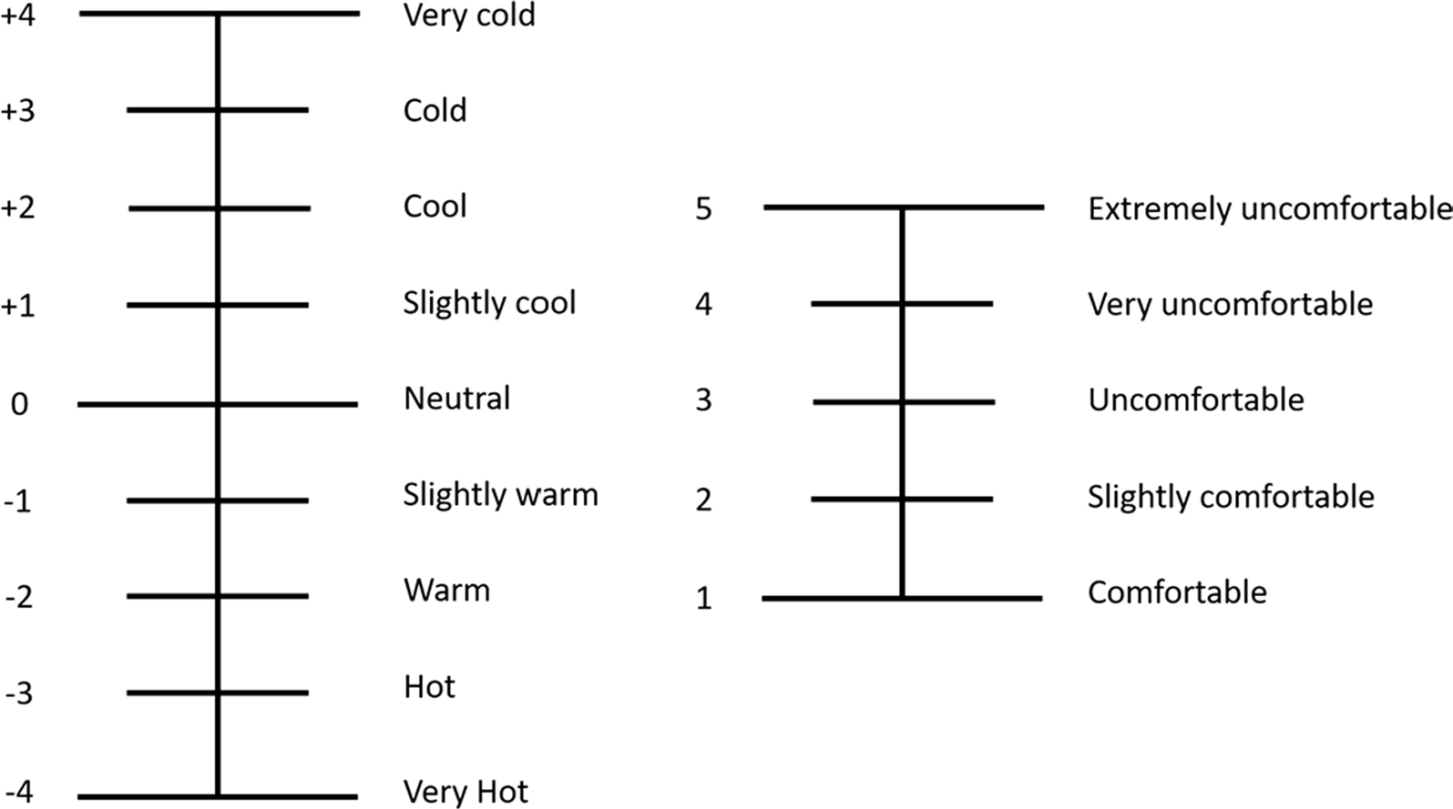
Scales used to assess thermal sensation and thermal comfort during exercise

During exercise, sweat is produced to allow for evaporative heat loss but, in turn, increases sensations of skin wettedness and stickiness which negatively affect overall comfort [8, 9, 56]. Hence, fabrics and clothing with superior wicking capabilities are essential for comfort during exercise [11, 57]. Thermal comfort and thermal sensation are the measures most used for assessing the perceptual effect of sports clothing during exercise (see Table 2). Thermal comfort is a subset of overall comfort [8] and is dependent on physical activity, environmental conditions, and material properties [58], whereas thermal sensation assesses satisfaction of the climatic conditions perceived by an individual [59]. In the current body of the literature, nine-point scales are commonly used to assess thermal sensation, whilst thermal comfort is commonly assessed via five-point scales (see Fig. 1). However, it should be noted that the descriptors within the scales, and the size of the scales used, vary across studies with some research using scales as large as 31 points and 13 points to assess thermal sensation and thermal comfort, respectively [56, 60]. Many studies have noted no significant differences in thermal sensation or thermal comfort during exercise when comparing synthetic and natural fibres [14, 16, 20, 27–30, 32, 33, 36, 43]. Conversely, others have reported significant improvements in thermal sensation [34, 46, 61] and thermal comfort [46] when comparing cotton to synthetic fibres [34], and when comparing semi-nude designs to full-body synthetic clothing designs [46, 61]. Manipulating material properties via chemical treatments or different knit structures and fits may improve the effect of synthetic materials on thermal sensation and thermal comfort. For example, thermal sensation was significantly improved when exercising in synthetic t-shirts using medium (3 mm) and larger (3.5 mm) knit sizes compared to small knit sizes (2 mm) in a hot and humid climate [49]. Thermal comfort is significantly improved in loose-fitting compared to tight fitting synthetic sports clothing [45]. Additionally, polyester t-shirts treated with PCM significantly improved thermal comfort during prolonged exercise in hot and humid conditions when compared to untreated polyester t-shirts [22]. Variations in the scales used to evaluate thermal comfort and thermal sensation, and the lack of equidistance between the scale’s points, may contribute to the state of current results [62]. Moreover, parameters such as thermal comfort and thermal sensation are based on oversimplified assumptions [62] that fail to capture intricate detail pertaining to the four main considerations of comfort outlined by Ozdil and Anand [25].

**Table 2:** Research findings from the assessment of thermoregulation, performance, and comfort in sportswear

Study	Sample size	Training status	Acclimatisation status	Sex	Apparel	Thermal	Physiological	Perception	PRFM
Abdallah et al. [17]	20	Trained	Not specified	Males and females	Long sleeve shirt and full-length pants CTR (synthetic blend) EXP (synthetic blend)	↔ T_core_ ↔ T_skin_ ↔ SR ↔ SL ↔ SE ↔ HS	↔ HR ↔ VO_2_ ↔ VT	↔ Thermal sensation ↔ Skin wettedness ↔ Clothing comfort ↔ RPE	↔ TTE
Barwood et al. [42]	8	Recreationally active	Not specified	Males	Lower-body garment CTR (shorts) EXP1 (correct fit) EXP2 (oversized)	↔ T_core_ ↔ T_skin_ ↔ T_body_ ↔ SL	↔ HR	↔ Thermal sensation ↔ Thermal comfort↔ RPE	↔ TT (5 km)
Brazaitis et al. [27]	8	Recreationally active	Not specified	Males	Long sleeve t-shirt, CTR (natural fibre) EXP (synthetic fibre)	↔ T_core_ ↔ T_skin_ ↔ T_body_ ↔ SL ↔ PSI ↑SE	↔ HR	↔ Thermal sensation ↔ clothing wetness	–
Collins et al. [55]	11	Recreationally active	Not specified	Females	Short sleeve t-shirts and sleeveless shirts CTR (synthetic and natural fibres) EXP (synthetic fibre)	↔ T_skin_ ↔ SR	↔ HR	↑Breathability ↔ Clinginess ↔ Freshness ↔ Weight ↓Dampness ↔ Smoothness ↑Softness ↔ Comfort ↔ RPE	–
Corbett et al. [19]	6	Trained	Not specified	Males	Clothing ensembles CTR (shorts, synthetic fibre) EXP1 (shorts and short sleeve jersey, synthetic blend) EXP2 (shorts and short sleeve jersey, synthetic blend) EXP3 (long sleeve base layer, cycling jacket, full length pants and skull cap, synthetic blend)	↔ T_core_ ↑T_skin_ ↑T_body_ ↑SL ↑SR	↑HR ↔ VO_2_ ↔ SBF	↑Thermal sensation ↓Thermal comfort ↑RPE	↔ TT (10 km)
Davis et al. [20]	8	Trained	Not specified	Males	Short sleeve t-shirt CTR (natural fibre) EXP1 (synthetic fibre) EXP2 (natural fibre blend)	↔ T_core_ ↔ T_skin_ ↔ T_body_ ↓T_micro_ ↔ SL ↔ PSI	↔ HR	↔ Thermal sensation ↔ Skin wetness ↔ Clothing comfort ↔ RPE	-
De Sousa et al. [14]	10	Recreationally active	Not specified	Males	Short sleeve t-shirt CTR (natural fibre) EXP (synthetic fibre)	↓T_core_ ↔ T_skin_ ↔ SL ↔ SR	↔ HR	↔ Thermal sensation ↔ Sweating sensation ↔ RPE	–
Filingeri et al. [60]	9	Recreationally active	Not specified	Males	Short sleeve t-shirt CTR (synthetic fibre) EXP1 (shirt dampened and worn during last 15 min of trial) EXP2 (shirt dampened and worn for 20 min pre-exercise and then during exercise)	↔ T_core_ ↓T_skin_ ↓SL	↓HR	↓Thermal sensation ↑Thermal comfort ↓Wetness sensation ↓RPE	–
Gavin et al. [28]	8	Trained	Acclimated	Males	Clothing ensembles CTR (short sleeve t-shirt and shorts, natural fibre) EXP1 (racing swimsuit, synthetic fibre) EXP2 (short sleeve t-shirt and shorts, synthetic fibre)	↔ T_core_ ↔ T_skin_ ↔ T_body_ ↔ SL ↔ SE	↔ HR ↔ VO_2_ ↔ VT	↔ Thermal sensation ↔ Thermal comfort	–
Gonzales et al. [49]	10	Trained	Not acclimated	Males	Short sleeve jersey CTR (synthetic fibre, small knit) EXP1 (synthetic fibre, medium knit) EXP2 (synthetic fibre, large knit)	↓T_skin_	↔ HR	↓Thermal sensation ↔ RPE	–
Ha et al. [43]	8	Recreationally active	Not specified	Females	Long sleeve shirt and full-length pants CTR (synthetic fibre, low absorption, low air permeability) EXP1 (synthetic fibre, low absorption, high air permeability) EXP2 (natural fibre, high absorption, high air permeability)	↔ T_core_ ↑T_skin_ ↑T_body_ ↔ SR ↔ SL ↑HS ↑T_micro_ ↓RH_micro_	↔ HR	↔ Thermal sensation ↔ Clothing comfort ↔ Sweating sensation ↔ Humidity sensation ↔ Skin wetness	–
Ha et al. [36]	5	Recreationally active	Not specified	Females	Short sleeve t-shirt, long—sleeved working dress and full-length pants CTR (natural fibre) EXP (synthetic fibre)	↑T_core_ ↔ T_skin_ ↑T_body_ ↔ SR ↔ SL ↔ HS ↔ T_micro_ ↔ RH_micro_	↑HR	↔ Thermal sensation ↔ Clothing comfort ↔ Humidity sensation	–
Herten et al. [51]	11	Trained	Not specified	Males	Shirt CTR (natural fibre) EXP (synthetic blend)	↔ T_core_ ↑SL ↔ T_micro_ ↔ RH_micro_	↔ HR	↔ Thermal comfort ↔ Heat sensation ↔ Wetness sensation ↔ Weight	–
Kaplan et al. [21]	5	Trained	Not specified	Males	Long sleeve t-shirt CTR (natural fibre) EXP1 (synthetic fibre) EXP2 (synthetic fibre) EXP3 (synthetic/natural blend) EXP4 (modified synthetic fibre)	↔ T_skin_ ↔ T_micro_ ↓RH_micro_	↔ HR	↓Thermal comfort ↓Skin wetness	–
Kwon et al. [30]	7	Recreationally active	Not specified	Females	Long sleeve t-shirt and full-length pants CTR (natural fibre) EXP1 (natural blend) EXP2 (synthetic fibre)	↓T_core_ ↑T_skin_ ↑T_body_ ↑SL ↔ HS ↓T_micro_ ↑RH_micro_	↑HR	↔ Thermal sensation ↔ Clothing comfort ↔ Sweating sensation ↔ Skin wetness ↔ Humidity sensation	–
Laing et al. [16]	10	Trained	Acclimated	Males	Long sleeve upper body garment CTR (synthetic fibre) EXP1 (synthetic/natural blend) EXP2 (natural fibre)	↓T_core_ ↔ T_skin_ ↔ SL ↑RH_micro_	↔ HR	↔ Thermal sensation ↔ Thermal comfort ↔ Wetness sensation ↔ RPE	–
Leoz-Abaurrea et al. [33]	16	Recreationally active	Not specified	Males and females	Short sleeve upper body garment CTR (natural fibre) EXP (synthetic blend)	↔ T_core_ ↔ T_skin_ ↔ T_body_ ↔ SR ↔ HS	↔ HR ↔ VO_2_	↔ Thermal sensation ↔ Clothing wetness ↔ RPE	-
Leoz-Abaurrea et al. [32]	12	Recreationally active	Not acclimated	Males	Short sleeve upper body garment CTR (natural fibre) EXP (synthetic blend)	↔ T_core_ ↔ T_skin_ ↔ T_body_ ↔ SR ↔ SL	↔ HR ↔ VO_2_	↔ Thermal sensation ↔ Sweating sensation ↑Clothing wetness ↔ RPE	–
Leoz-Abaurrea et al. [34]	20	Recreationally active	Not specified	Males and females	Short sleeve upper body garment CTR (natural fibre) EXP (synthetic blend)	↑T_core_ ↔ SR ↔ SL	↑HR ↔ VO_2_ ↔ AP	↑Thermal sensation ↔ Sweating sensation ↔ Clothing wetness ↔ RPE	–
Lin et al. [63]	10	Recreationally active	Not specified	Females	Sports Bra CTR (synthetic and natural blend) EXP (synthetic and natural blend)	↔ T_core_ ↓T_skin_ ↔ SL	↔ HR	↔ Wetness ↔ Coldness ↔ Breathability ↔ Itchiness ↔ Softness ↔ Comfort	–
MacRae et al. [46]	12	Recreationally active	Not specified	Males	Full body garment CTR (shorts) EXP1 (synthetic blend—oversized) EXP2 (synthetic blend—correct fit)	↔ T_core_ ↑T_skin_ ↔ SR	↔ HR ↔ Q	↑Thermal sensation ↓Thermal comfort ↔ Skin wetness ↔ RPE	↔ TT /MPO (6 km)
McFarlin et al. [22]	14	Trained	Not specified	Males and females	t-shirt CTR (synthetic fibre) EXP (Modified synthetic fibre)	↓T_skin_	–	↑Thermal comfort ↓RPE	↑TTE
Raccuglia et al. [56]	8	Recreationally active	Not specified	Males	Short sleeve t-shirt CTR (synthetic fibre—high contact) EXP1 (synthetic fibre—medium contact) EXP2 (synthetic fibre—low contact)	↔ T_core_↔ SL	↔ HR	↔ Thermal sensation ↓Comfort ↔ Skin wetness ↑Stickiness	–
Roberts et al. [26]	7	Recreationally active	Not specified	Males	t-shirt CTR (bare chested) EXP1 (synthetic fibre) EXP2 (synthetic blend) EXP3 (natural fibre)	↑T_core_ ↑T_skin_ ↑SE	–	↑Thermal sensation ↓Thermal comfort ↔ RPE	–
Scholler et al. [18]	10	Trained	Not specified	Males	Full body racing suits EXP1 (synthetic blend, knit) EXP2 (synthetic blend, woven) EXP3-4 (synthetic blend, knit) EXP5-6 (synthetic blend, woven)	↔ T_core_ ↔ T_skin_	↔ HR	↑Thermal sensation (EXP1 vs EXP5) ↔ RPE	↔ PO (20 min at RPE 4/10)
Sperlich et al. [29]	8	Trained	Not specified	Males	t-shirt CTR (natural fibre) EXP1 (synthetic fibre, 4 channel) EXP2 (synthetic fibre, 6 channel) EXP3 (synthetic fibre, 8 channel)	↔ T_core_ ↔ T_skin_ ↔ T_body_ ↔ SL ↓RH_micro_	↔ HR ↔ VO_2_ ↔ VT ↔ BL	↔ Thermal sensation ↔ Sweating sensation ↔ Clothing wetness ↔ RPE	↔ TTE
Tsuji et al. [61]	10	Recreationally active	Not specified	Males	Long sleeve t-shirt and shirts CTR (shorts—synthetic fibre) EXP (long sleeve t-shirt—synthetic blend, shorts—synthetic fibre)	↔ T_core_ ↓T_skin_ ↓T_body_ ↑SL ↔ HS	↑HR ↔ SBF	↑Thermal sensation ↑RPE	–
Ueda et al. [15]	3	Recreationally active	Not specified	Males	Long sleeve t-shirts CTR (natural fibre, plain knit) EXP1 (natural fibre, stockinet) EXP2 (natural fibre, plain knit) EXP3 (natural fibre, jacquard knit) EXP4 (natural fibre, mesh knit)	↔ T_core_ ↔ T_skin_ ↔ SR ↔ T_micro_ ↔ RH_micro_	-	↑Comfort	–
Varadaraju et al. [50]	10	Recreationally active	Not specified	Males	Short sleeve t-shirt (synthetic fibre) CTR (tricot knit) EXP1-3 (open knit, modified)	↓T_skin_ ↓RH_micro_	↓HR	↑Comfort ↓Skin temperature ↓Skin moisture	–
Wang et al. [77]	8	Recreationally active	Not specified	Males	Upper body garment CTR (natural fibre) EXP1 (body mapping sportswear) EXP2 (EXP1 modified)	↓T_core_ ↓T_skin_ ↓T_body_ ↔ SL ↔ SE	↓HR ↔ VO_2_	↓Thermal sensation ↓Skin humidity ↑Comfort sensation ↔ RPE	–
Wingo et al. [81]	9	Recreationally active	Not specified	Males	Long sleeve t-shirt CTR (bare chested) EXP1 (synthetic fibre) EXP2 (natural fibre)	↔ T_core_ ↑T_skin_ ↑SR ↔ SL	↔ HR	↑Thermal sensation ↔ RPE	–
Zhang et al. [45]	8	Recreationally active	Not specified	Males	9 Short sleeve t-shirts (natural fibres, differed in neck and hem opening size)	↔ T_core_ ↔ T_skin_ ↔ T_micro_ ↔ RH_micro_ ↔ SL ↔ HS ↔ SE	↔ HR	↔ Thermal sensation ↑Thermal comfort (loose fit vs tight fit) ↔ Skin wetness	–

*PRFM* performance, *CTR* control, *EXP* experimental, *Tcore* core temperature, *Tskin* skin temperature, *Tbody* body temperature, *SR* sweat rate, *SL* sweat loss, *SE* sweat evaporated, *HS* heat storage, *Tmicro* microclimate temperature, *RHmicro* microclimate relative humidity, *PSI* physiological strain index, *HR* heart rate, *VO*_*2*_ oxygen consumption, *VT* ventilation, *SBF* skin blood flow, *BL* blood lactate, *AP* arterial pressure, *Q* cardiac output, *TTE* time to exhaustion, *TT* time trial, *PO* power output, *MPO* mean power output, − not assessed, ↑ significantly higher than control, ↓ significantly lower than control, ↔ no significant difference

Davis and Bishop [24] proposed the utilisation of various comfort parameters to gain a more comprehensive understanding of clothing comfort during exercise. Using a multi-dimensional approach that incorporates the four main considerations outlined by Ozdil and Anand [25] could provide better insights into the important design features and properties of sports clothing that relate to wearer comfort. Three recent studies by Collins et al. [55], Raccuglia et al. [56], and Lin et al. [63] included additional parameters assessing the sensorial and thermo-physiological considerations of comfort including breathability [55, 63], clinginess/stickiness [55, 56], freshness [55], weight [55], dampness [55], smoothness [55], softness [55, 63], itchiness [63], and overall texture sensation [56]. Raccuglia et al. [56] determined that short sleeve synthetic t-shirts with reduced moisture absorption capacity (172 gm^2^) were significantly more sticky, rough, and uncomfortable when compared to an identical synthetic t-shirt with higher moisture absorption capacity (278 gm^2^). Additionally, an increase in stickiness sensation was the strongest predictor of discomfort after 30 min of moderate intensity exercise (*r*^2^ = 0.59, *p* = 0.001) in a warm environment (27.4 °C, 49.4% RH) [56]. Lin et al. [63] observed no significant differences in coldness, breathability, itchiness, softness, or comfort sensations when comparing a single jersey bra (35% polyester, 8% lycra, 57% cotton) to a sports bra of identical fibre composition with dynamic water pumping fabrics in females during light intensity exercise. Finally, Collins et al. [55] noted significant improvements in breathability, dampness, and softness in female subjects wearing lightweight synthetic sports apparel (54% nylon, 40% polyester, 3% elastane, 3% X-static® nylon) compared to the subjects’ preferred exercise apparel (including an array of polyester and cotton fibre clothing). However, no improvements in overall comfort during exercise were observed [55]. Though these three studies used short exercise durations (i.e. < 30 min) and relatively mild climates, their multidimensional assessment of clothing comfort generates useful insights pertaining to three of the four considerations of comfort (i.e. sensorial, body-movement, and thermo-physiological) outlined by Ozdil and Anand [25]. Future research should aim to assess overall comfort during exercise using a multidimensional approach that incorporates parameters capturing the four main considerations of clothing comfort. Additional efforts should be made to investigate the relationship between measures of comfort, material properties, and physiological data to understand their interplay and how they collectively contribute to exercise performance. Such work may inform the design of more effective sports clothing to enhance comfort, thermoregulation, and performance and aid in the development of comprehensive clothing recommendations for recreational exercisers and elite-level athletes.

## Sex and the Assessment of Sports Clothing, Comfort, and Thermoregulation

Research has compared the thermoregulatory and physiological differences between males and females during exercise. Though neither males nor females have an inherent advantage regarding thermoregulation [64], males tend to have a higher skin temperature to core temperature gradient which allows for greater heat exchange away from the core [65]. In contrast, females tend to have a higher surface area to mass ratio [65, 66] which may be beneficial to thermoregulation as the ratio of evaporatively cooled body surface to metabolically active tissue is greater [65]. Females may also generate less heat and require less sweat gland output than males given their smaller body size and lower muscle mass [64]. This is supported by Gagnon and Kenny [67] who observed significantly lower sweat gland output in females on the upper back, chest, and forearm during vigorous exercise in hot and dry conditions (40 °C, 24% RH). However, equal amounts of sweat loss (Δ body mass [g]) were identified in males and females during vigorous intensity exercise in moderate heat and humidity (25.5 °C, 53% RH), despite significantly higher sweat rates (g m^−2^ h^−1^) at the mid lateral back, mid front, and sides of the upper body in males [68]. These results suggest that males may benefit from wearing sports clothing with less skin coverage on the upper body to promote sweat evaporation. Unlike males, females experience fluctuations in body temperature and thermoregulatory responses during exercise over the menstrual cycle due to the influence of reproductive hormones [64, 69–71]. Whilst thermoregulatory fluctuations can be regulated by oral contraceptives [70], increased levels of skin blood flow and sweat secretion have been noted at higher core temperatures in the luteal phase [70, 72, 73]. These results suggest that females in the luteal phase may benefit from wearing sports clothing with optimal wicking capabilities or less skin coverage to promote sweat evaporation and convective cooling effects during exercise. Importantly, the assessment of clothing on thermoregulation, comfort, and performance during exercise in female subjects should control for menstrual cycle phase and hormonal contraceptive use to minimise their impact on results.

Studies investigating the impact of sports clothing on thermoregulation, comfort, and performance during exercise have primarily included male subjects only (72%), whilst a small proportion of studies have exclusively included females (16%), and both males and females (12%) (see Table 2). Of the nine studies including female subjects, seven controlled for the effect of menstrual cycle on thermoregulation [17, 30, 33, 34, 36, 43, 63]; however, five of these studies provided very little detail as to how menstrual cycle phase or use of hormone contraceptives was screened or controlled [30, 34, 36, 43, 63]. Of the four studies including males and females, none of them provided a direct comparison between the sexes nor reported sex-specific results. Additionally, of the five studies exclusively including females, none of them included trained female athletes. This is a critical limitation considering that endurance-trained individuals can tolerate higher levels of heat stress via physiological adaptations including reduced core and skin temperatures at rest and during exercise, increased plasma volume, decreased sweat onset temperature, and increased whole body sweat rate [74]. Ha et al. [36] and Kwon et al. [30] identified significant increases in core temperature and heart rate in recreationally active females wearing synthetic clothing during light intensity exercise in moderate heat and humidity (24–30 °C, 50% RH) compared to clothing comprised of natural fabrics. Though this research suggests thermoregulation in females is impaired by synthetic materials, both studies required participants to exercise in clothing with large amounts of body coverage and high levels of insulation (see Table 1). Leoz-Abaurrea and Aguado-Jimenez [34] observed significant increases in core temperature, heart rate, and thermal sensation in a pooled sample of males and females (*n* = 20) wearing a synthetic upper-body compression garment during exercise compared to a t-shirt comprised of natural fibres. In contrast, Leoz-Abaurrea et al. [32] replicated the study exclusively in males and observed no significant differences in the same parameters despite an exercise duration ~ 26 min longer than that by Leoz-Abaurrea and Aguado-Jimenez [34]. This comparison may suggest the sports clothing examined across both studies provided a less favourable impact on thermoregulation and comfort for the female subjects in the Leoz-Abaurrea and Aguado-Jimenez [34] study. Clearly, the interaction between sex and clothing on thermoregulation, comfort, and performance during exercise needs to be explored. Such research could provide an explanation for this phenomenon and generate insight into the important characteristics required in the design of sports clothing to maximise thermoregulation and comfort in males and females separately given their thermo-physiological differences.

## Simulating ‘Real Environments’ in Laboratory Settings

Thermoregulatory demands on athletes can vary substantially between outdoor competition and laboratory-based testing, even when attempting to match environmental conditions [75]. Though outdoor trials can mimic real competition without compromising the test reliability provided by a laboratory setting [76], their feasibility may be compromised by logistical obstacles such as unpredictable changes in weather conditions or the availability of portable testing equipment. Nevertheless, it is critical that a range of factors are considered when designing valid testing protocols to examine the effect of sports clothing during exercise in the heat. These may include exercise intensity and trial type, exercise duration, and environmental conditions including ambient temperature and RH. The following section critically evaluates the exercise protocols and environmental conditions used across the current body of the literature, explores their influence on results and conclusions, and identifies important directions for future research in this space.

### Exercise Intensity and Trial Type

An array of exercise intensities are employed to assess sports clothing and its effect on thermoregulation, comfort, and exercise performance (see Table 3). 20 out of 32 studies employed light-to-moderate intensities (20–60% VO_2_ peak) [14, 15, 18–21, 27, 30, 32–34, 36, 43, 45, 49, 51, 56, 61, 63, 77] as characterised by American College of Sports Medicine exercise guidelines [78]. Of the 20 studies, only five identified significant differences in core temperature when comparing synthetic and natural fabrics during exercise [14, 30, 34, 36, 77]. Additionally, only two studies observed significant differences in skin temperature [30, 77], one study identified significant differences in sweat loss [30], and one study reported significant differences in thermal sensation [34] when comparing synthetic and natural fabrics. Though De Sousa et al. [14] employed hot and humid conditions (33 °C, 60% RH) and showed fabric-dependent differences in core temperature, a similar study by Leoz-Abaurrea et al. [32] found no significant differences in core temperature when comparing synthetic and natural fabrics despite using a longer exercise protocol (~ 10 min) of the same intensity in hotter but less humid conditions (39.9 °C, 35% RH). Other studies using light-to-moderate intensities noted significant increases (0.27–1.06%) in core temperature during exercise when synthetic compared to natural fabrics were worn [30, 34, 36]. However, these studies required participants to exercise in compression clothing [34], and in long sleeve t-shirts and full length pants which promote insulation via increased body coverage [30, 36]. Significant reductions in skin temperature were identified for synthetic fabrics during exercise of light [19, 43, 61, 63] and moderate [45, 49, 61] intensities in mild to warm temperatures (14.5–30 °C). But of the 17 studies identified in this review that assessed skin temperature during light-to-moderate intensities, ~ 50% of them did not identify significant differences in skin temperature when comparing different clothing designs and fabrics [14, 15, 18, 20, 21, 27, 32, 33, 36]. Though skin temperature is universally measured in clothed and nude areas across the literature, it should be noted that some of these studies only assessed skin temperature at ≤ 4 individual sites on the body [14, 15, 21, 32, 33] compared to ≥ 6 sites in those that identified significant differences [19, 43, 45, 61]. Nevertheless, the most common finding amongst those using light-to-moderate intensities, irrespective of the fabric and clothing ensembles assessed, is a lack of significant differences in core temperature, heart rate, oxygen consumption (VO_2_), sweat loss, thermal sensation, and thermal comfort (see Table 2). Whilst these results suggest that no particular fabric or design is superior to another in regards to thermoregulation or comfort during exercise, it is possible that the light-to-moderate exercise intensities in previous studies do not induce sufficient thermo-physiological and perceptual strain to adequately assess the capacity of the clothing to aid thermoregulation or improve comfort [3].

**Table 3:** Exercise and environmental conditions in the assessment of thermoregulation, performance, and comfort in sportswear

Study	Ambient temperature (°C)	Relative humidity (%)	Heat Index (°C)	Air flow (m^.^s^−1^)	Exercise intensity	Exercise duration	Exercise mode
Abdallah et al. [17]	24.5	23	24	–	MPO (85%)	~ 14 min	Fixed rate/incremental (cycle ergometer)
Barwood et al. [42]	35.2	55	43	–	Treadmill speed (10 km/h and 12 km/h)	15 min (+ 5 km TT)	Fixed rate/self-regulated (treadmill run)
Brazaitis et al. [27]	25	60	25	0.3	Treadmill speed (8 km/h)	60 min (3 × 20-min trials, 5 min rest between trials)	Fixed rate (treadmill run)
Collins et al. [55]	18	–	–	–	RPE (17/20)	20 min	Incremental (treadmill run)
Corbett et al. [19]	14.5	46.8	13	3.6, 8.3, and 16.7	PPO (35–55%)	95 min (+ 10 km TT)	Fixed rate/self-regulated (cycle ergometer)
Davis et al. [20]	32	32.5	31	3.1	VO_2_ peak (60%)	45 min	Fixed rate (treadmill run)
De Sousa et al. [14]	33	60	40	-	VO_2_ peak (50%)	45 min	Fixed rate (cycle ergometer)
Filingeri et al. [60]	30	44	30	2.0	VO_2_ max (70%)	25 min	Fixed rate (treadmill run)
Gavin et al. [28]	30	35	29	3.1	VO_2_ max (70%)	30 min	Fixed rate (treadmill run)
Gonzales et al. [49]	29	71	33	2.8	Power Output (150 W)	15 min	Fixed rate (cycle ergometer)
Ha et al. [43]	27	50	27	0.1	VO_2_ max (30%)	40 min	Fixed rate (cycle ergometer)
Ha et al. [36]	24	50	24	0.1	VO_2_ max (30%)	40 min	Fixed rate (cycle ergometer)
Herten et al. [51]	25	50	25	-	VO_2_ max (50% and 60%)	60 min (30 min at each VO2 max	Fixed rate/incremental (treadmill walk)
Kaplan et al. [21]	24	60	24	-	Treadmill speed (6 and 9 km/h)	30 min (15 min at each speed)	Fixed rate/incremental (treadmill walk)
Kwon et al. [30]	30	50	31	1.5	VO_2_ max (40%)	60 min	Fixed rate (cycle ergometer)
Laing et al. [16]	32	20	30	3.1	VO_2_ max (70%)	30 min	Fixed rate (treadmill run)
Leoz-Abaurrea et al. [33]	22.7	59	23	2.5	VO_2_ peak (50%)	56 min (4 × 14 min trials, 1 min rest between trials)	Fixed rate (cycle ergometer)
Leoz-Abaurrea et al. [32]	39.9	35	45	2.5	VO_2_ peak (50%)	56 min (4 × 14 min trials, 1 min rest between trials)	Fixed rate (cycle ergometer)
Leoz-Abaurrea et al. [34]	39.9	34	45	2.5	VO_2_ max (50%)	30 min	Fixed rate (cycle ergometer)
Lin et al. [63]	27	46	27	–	Treadmill speed (7 km/h)	20 min	Fixed rate (treadmill run)
MacRae et al. [46]	24	60	24	2.0	VO_2_ peak (60%)	60 min (+ 6 km TT)	Fixed rate/self-regulated (cycle ergometer)
McFarlin et al. [22]	35	55	43	1.9	PSI (5, 7.5, 9)	~ 45 min	Incremental/self-regulated (treadmill run)
Raccuglia et al. [56]	28	49.4	28	0.2	Treadmill speed (10.2 km/h)	30 min	Fixed rate (treadmill run)
Roberts et al. [26]	20.6	47.5	20	3.0	Treadmill speed (6, 12, and 15 km/h)	46 min (2 × 23 min interval session, ~ 8 min at each speed per interval, 70 s rest between intervals)	Incremental (treadmill run)
Scholler et al. [18]	33	65	41	1.2	RPE (4/10)	20 min	Fixed rate/self-regulated (cycle ergometer)
Sperlich et al. [29]	31.7	42	32	0.3	VO_2_ max (70%)	30 min (+ TTE)	Fixed rate/self-regulated (treadmill run)
Tsuji et al. [61]	30	60	33	0.8	VO_2_ peak (20% and 50%)	120 min (3 × 20 min intervals at each VO2 peak, 5 min rest between intervals)	Fixed rate (cycle ergometer)
Ueda et al. [15]	25	50	25	0.3	VO_2_ max (30% and 45%)	~ 30 min	Fixed rate/incremental (treadmill run)
Varadaraju et al. [50]	33	60	40	0.15	Treadmill speed (12 km/h)	40 min	Fixed rate (treadmill run)
Wang et al. [77]	30	40	30	0.2	Treadmill speed (5 and 10 km/h)	60 min (40 min at 5 km/h, 20 min at 10 km/h)	Fixed rate (treadmill run and walk)
Wingo et al. [81]	22	26	21	2.4	VO_2_ peak (65%)	45 min	Fixed rate (treadmill run)
Zhang et al. [45]	25	50	25	0.2	VO_2_ max (55%)	30 min	Fixed rate (treadmill run)

*WBGT* wet bulb globe temperature, *MPO* maximal power output, *VO*_*2*_ oxygen consumption, *TT* time trial, *RPE* rate of perceived exertion, *PPO* peak power output, *TTE* time to exhaustion, *PSI* physiological strain index

Previous studies have highlighted the need to employ vigorous intensities (> 60% VO_2_ peak) to better understand the effect of clothing during exercise as it may induce the thermo-physiological and perceptual strain often experienced by athletes during competition [24, 79]. In fact, most endurance events require athletes to maintain moderate-to-vigorous exercise intensities via self-regulation and pacing strategies to ensure they cover a set amount of distance as quickly as possible [76, 80]. To date, only nine studies have employed vigorous exercise intensities (see Table 3). These investigations have shown significant differences in core temperature [16, 26, 81], skin temperature [22, 26, 60], sweat evaporation [26, 28], heart rate [60], and microclimate humidity [29] when comparing various clothing ensembles and fabrics. This suggests the impact of clothing on thermo-physiological strain is different at vigorous exercise intensities, confirming the need to conduct well-controlled studies in ecologically valid settings. Six of these studies utilised fixed-intensity exercise protocols which minimise test–retest variation and allow researchers to measure physiological markers with less difficulty compared to incremental exercise trials as they mitigate fluctuations in exercise intensity and the changes within data that may occur as a result [76]. However, exercise performance cannot be measured using fixed-intensity exercise alone. Fixed-intensity protocols used within the current literature consist of 10–30 min of exercise at intensities of 20–50% VO_2_ peak (see Table 3) and are likely inadequate to simulate the demands of endurance competition. To address these limitations, recent research has employed self-paced exercise tests immediately following moderate-to-vigorous fixed-intensity protocols in both elite [17, 19, 29] and recreationally active [42, 46] populations. This may be effective given self-paced time trials of a set distance are valid tests of performance in elite athletes as they allow subjects to maintain moderate-to-vigorous intensities similarly to competition [76]. Additionally, test–retest variation in distance-based time trials can be reduced if well-trained subjects are recruited and familiarisation protocols are prescribed [76].

### Exercise Duration

Long-distance endurance events typically exceed 120 min in duration. To date, two studies have utilised light-intensity exercise protocols of 120 min when assessing clothing, thermoregulation, and comfort [19, 61]. Both studies generated significant differences in skin temperature, sweat loss, thermal sensation, and RPE across multiple clothing types, fabric compositions (both synthetic and natural), and body coverage (full body and semi-nude) [19, 61]. This may suggest that exercise protocols of 120 min in duration may induce enough thermoregulatory strain to adequately assess the effect of clothing during exercise irrespective of exercise intensity. However, the logistics of designing an exercise protocol incorporating moderate-to-vigorous intensities of this length may not be feasible in most research settings. Most studies assessing sports clothing during exercise in the heat utilised protocols of 10–45 min in duration (see Table 3) with mixed results (see Table 2). However, studies using exercise protocols of 45 min have also highlighted the need for longer exercise bouts to detect significant differences amongst clothing designs [14, 20, 81]. Moreover, this may suggest that sports clothing may only provide thermal, perceptual, and performance benefits during long duration and high intensity exercise bouts, though further research incorporating such protocols is warranted to confirm this. Recent studies have also prescribed moderate and vigorous intensities during 60-min exercise bouts [46, 51]. MacRae et al. [46] prescribed a 60 min protocol at 65% VO_2_ peak followed by a 6 km time trial but observed no significant differences in thermoregulatory, physiology, comfort, or performance-based measures when comparing correctly fitted (11–15 mmHg) and oversized (8–13 mmHg) compression garments. Herten et al. [51] compared a blended synthetic t-shirt to a purely synthetic t-shirt (100% polyester) using two consecutive 30-min trials performed at 50% and 60% VO_2_ peak but only observed significant differences in absolute and relative sweat loss and not in core temperature, heart rate, or thermal comfort. Though these results may indicate the need for exercise protocols to exceed 60 min to sufficiently examine the capacity of clothing to aid thermoregulation, comfort, and performance, the environmental conditions were mild in both studies (24–25 °C, 50–60% RH) [46, 51]. Clearly, the feasibility, ethical, and logistical challenges of designing appropriate exercise protocols for the assessment of sports clothing during exercise in the heat should be considered. However, it appears that high-intensity exercise protocols may need to be 45–60 min in duration to mimic actual training and competition demands when assessing sports clothing in endurance athletes.

### Environmental Conditions

Ambient temperature and RH are the most modified environmental conditions when assessing clothing during laboratory exercise trials. To date, studies have used a range of environmental conditions (18–40 °C, 20–60% RH) which has led to mixed results. Studies comparing synthetic and natural fabrics using hot and dry conditions (30–32 °C, 20–42% RH) observed no significant differences in core temperature [20, 28, 29], skin temperature [16, 20, 28, 29], sweat loss [16, 20, 28, 29], heart rate [16, 20, 29], thermal sensation [16, 20, 28, 29], thermal comfort [16, 28], or exercise performance [28, 29]. In contrast, studies that used hot and humid conditions (29–35 °C, 50–71% RH) have reported significant differences in core temperature [14, 30], skin temperature [22, 30, 49, 54], sweat loss [30], thermal sensation [18, 49], and exercise performance [22] when comparing across multiple fabrics and designs. The contrasting results between studies using hot and dry compared to hot and humid conditions may be expected given that RH plays a more crucial role in dictating the level of heat that can be dissipated via sweat evaporation [8, 35, 47]. However, the interplay between temperature and RH appears to be more complex; simply increasing one of these environmental parameters at the expense of the other may not be enough to increase the thermo-physiological and perceptual strain during exercise. Studies that used warm temperatures (23–25 °C) and high humidity (50–60%) observed no significant differences in core temperature [27, 33, 46, 51], skin temperature [21, 27, 33], sweat rate [33], heart rate [21, 27, 33, 46, 51], thermal sensation [27, 33, 51], and RPE [33, 46] when comparing synthetic and natural fabrics, and blended materials. Similarly, studies using hotter ambient temperatures (> 35 °C) and low humidity (34–35%) reported no significant differences in core temperature [32], skin temperature [32], sweat rate [32, 34], sweat loss [32, 32, 34, 34], thermal sensation [32], or RPE [32, 34]. These findings support previous literature and indicate a need for protocols which simultaneously implement hotter and more humid conditions to explore the full capacity for sports clothing to aid thermoregulation, maximise comfort, and enhance performance. These conditions are also important for ecological validity since endurance athletes often compete in hot and humid climates [3].

Simply increasing temperature and RH beyond 30 °C and 60%, respectively, may address the inconsistencies and limitations of previous research. However, there are currently no definitive classifications for determining the severity of environmental conditions that should be prescribed to safely induce sufficient levels of thermo-physiological and perceptual strain in exercise research. Heat index is a scale globally used to determine the risk of heat illness and is calculated using ambient temperature (°C) and RH (%). Though many heat index equations exist, Anderson et al. [82] concludes that values calculated from one algorithm are correlated well with those from other algorithms. Nevertheless, the equation utilised by the National Oceanic and Atmospheric Administration (NOAA) [83] was used to produce the heat index values presented in Table 3. Heat indexes < 26.6 °C present no major risk of heat-related health concerns, and whilst heat indexes of 26.7–32.2 °C present a moderate heat stress risk, prolonged exercise can safely continue if participants are continuously monitored and given hydration breaks every 30 min [84]. Exercise can continue in conditions eliciting a heat index between 32.3 and 39.4 °C though extreme caution is advised; participants should be sufficiently hydrated prior to exercise and be provided with hydration breaks frequently throughout exercise, and exercise should not exceed 2 h [84]. Finally, exercising in conditions eliciting a heat index > 39.4 °C presents considerable danger and should be cancelled and/or postponed to a cooler time of day [84].

Twenty-two studies in this review utilised environmental conditions producing a heat index ≤ 32.2 °C [15–17, 19–21, 26–30, 33, 36, 43, 45, 46, 51, 56, 60, 63, 77, 81]. Though the level of thermo-physiological and perceptual strain provided from the climate alone may have been low-moderate, many implemented exercise bouts of high intensity [46, 56, 60, 77, 81] and long exercise durations in recreationally active participants [26, 27, 30, 33, 36, 43, 46, 77, 81]. In contrast, of the 11 studies that recruited endurance-trained subjects, eight used environmental conditions ≤ 32.2 °C [16, 17, 19–21, 28, 29, 51], only one study used environmental conditions eliciting a heat index between 32.3 and 39.4 °C [49]**,** and only three studies confirmed whether or not the subjects were heat acclimated [16, 28, 49]. Importantly, most of these studies showed no significant differences in core temperature [17, 19, 20, 28, 29, 51], skin temperature [16, 17, 20, 21, 28, 29], sweat loss [16, 17, 20, 28, 29, 49], exercise performance [17, 19, 28, 29], thermal sensation [16, 17, 20, 28, 29, 51], and thermal comfort [16, 17, 28, 51] when comparing across multiple fabrics and designs. This may suggest the chosen environmental conditions were insufficient in inducing enough thermo-physiological and perceptual strain in trained subjects to determine the impact of the clothing during exercise. However, it is also possible that the sports clothing and the control garments assessed in these studies had an equal impact on thermoregulation, comfort, and performance. Nevertheless, further research is warranted to explore the capacity for sports clothing to aid thermoregulation, comfort, and performance in trained subjects using hotter and more humid conditions, specifically those equal to a heat index between 32.3 and 39.4 °C. However, such conditions must be considered in conjunction with exercise intensity and duration to ensure participant safety.

## Conclusion

Thermoregulation is the process of regulating body temperature and is vital for maintaining athlete health and performance during exercise in the heat. Sports clothing aims to mitigate the insulative effect of clothing to promote heat loss via sweat evaporation, whilst maintaining optimal comfort for the wearer. To date, most studies have utilised light-to-moderate exercise intensities, short and varied exercise durations, and a multitude of environmental conditions to assess the impact of sports clothing on comfort, performance, and thermoregulation. The disparities across methodologies and the insufficient thermo-physiological and perceptual strains applied have led to mixed findings. Future studies assessing sports clothing in athletic populations should consider using prolonged fixed-intensity protocols (i.e. ≥ 45 min) of vigorous intensities (i.e. ≥ 60% VO_2_ peak) in hot and humid climates (i.e. heat index 32.3–39.4 °C) to ensure testing conditions reflect competition demands regularly experienced by endurance athletes. Self-paced tests such as distance-based time trials are recommended following fixed-intensity protocols to assess exercise performance. It is important to carefully consider rest and/or hydration periods to ensure participant safety. Despite known physiological differences [65], no research has compared the effect of clothing and fabrics between male and female athletes during exercise in the heat. When doing so, studies should control for potential confounders such as menstrual cycle phase and hormonal contraceptive use which have the potential to influence thermo-physiological responses at rest and during exercise. Comfort is a complex sensation that has been predominantly assessed via rudimentary scales including thermal comfort and thermal sensation which fail to capture psychological, sensorial, body-movement, and thermo-physiological considerations. Future studies should employ a multidimensional approach to comfort assessment by including additional parameters such as breathability, softness, clinginess, and wetness to holistically evaluate their impact on the wearer during exercise. An extensive battery of material tests should be completed to ascertain a clothing ensemble's water vapour permeability as well as its evaporative and overall heat loss capacity. Such information may add to the level of understanding provided from generic parameters including knit structure, mass, and air permeability and complement or support the results obtained from human trials. Finally, future research should continue to focus on the development and assessment of new sports clothing designs and chemically treated fibres to better understand which fabric composition or treatment optimises thermoregulation, comfort, and performance during exercise in the heat.

## Data Availability

Not applicable.

## Abbreviations

AP: Arterial pressure
BL: Blood lactate
CSR: Centre for Sport Research
CTR: Control
EXP: Experimental
HR: Heart rate
HS: Heat storage
MPO: Mean power output
NOAA: National Oceanic and Atmospheric Administration
PCM: Phase change material
PO: Power output
PRFM: Performance
PSI: Physiological strain index
Q: Cardiac output
RH: Relative humidity
RH_micro_: Microclimate relative humidity
RPE: Rate of perceived exertion
SBF: Skin blood flow
SE: Sweat evaporated
SL: Sweat loss
SMA: Sports Medicine Australia
SR: Sweat rate
*T*_body_: Body temperature
*T*_core_: Core temperature
*T*_micro_: Microclimate temperature
*T*_skin_: Skin temperature
TT: Time trial
TTE: Time to exhaustion
VO_2_: Oxygen consumption
VO_2_ max / peak: Maximal oxygen consumption
VT: Ventilation
